# Advanced Imaging for the Early Diagnosis of Local Recurrence Prostate Cancer after Radical Prostatectomy

**DOI:** 10.1155/2014/827265

**Published:** 2014-03-13

**Authors:** Valeria Panebianco, Flavio Barchetti, Daniela Musio, Francesca De Felice, Camilla Proietti, Elena Lucia Indino, Valentina Megna, Orazio Schillaci, Carlo Catalano, Vincenzo Tombolini

**Affiliations:** ^1^Department of Radiological Sciences, Oncology and Pathology, Sapienza University of Rome, 00161 Rome, Italy; ^2^Department of Diagnostic and Molecular Imaging, Interventional Radiology, Nuclear Medicine and Radiation Therapy, University Hospital “Tor Vergata”, 00133 Rome, Italy; ^3^Department of Radiological Sciences, Oncology and Pathology, Spencer-Lorillard Foundation, Sapienza University of Rome, 00161 Rome, Italy

## Abstract

Currently the diagnosis of local recurrence of prostate cancer (PCa) after radical prostatectomy (RT) is based on the onset of biochemical failure which is defined by two consecutive values of prostate-specific antigen (PSA) higher than 0.2 ng/mL. The aim of this paper was to review the current roles of advanced imaging in the detection of locoregional recurrence. A nonsystematic literature search using the Medline and Cochrane Library databases was performed up to November 2013. Bibliographies of retrieved and review articles were also examined. Only those articles reporting complete data with clinical relevance for the present review were selected. This review article is divided into two major parts: the first one considers the role of PET/CT in the restaging of PCa after RP; the second part is intended to provide the impact of multiparametric-MRI (mp-MRI) in the depiction of locoregional recurrence. Published data indicate an emerging role for mp-MRI in the depiction of locoregional recurrence, while the performance of PET/CT still remains unclear. Moreover Mp-MRI, thanks to functional techniques, allows to distinguish between residual glandular healthy tissue, scar/fibrotic tissue, granulation tissue, and tumour recurrence and it may also be able to assess the aggressiveness of nodule recurrence.

## 1. Introduction

Currently most of the patients (about 50%) affected by prostate cancer (PCa) are treated with radical prostatectomy (RP) [1]. This is due to good functional results regarding oncological radical criteria that can be achieved thanks to modern surgical techniques. Nevertheless, local relapse after RP is a crucial point due to its high frequency. Generally, PSA is a nonspecific tumor marker but, after RP, the rise of PSA serum levels means that PSA producing tissue remained and it suggests the presence of persistent or recurrent disease in the pelvis or distant metastases. Moreover, a persistently elevated PSA serum level could be also due to residual glandular healthy tissue [2]. Tumor recurrence is usually preceded by the rise of PSA serum values and occurs in 20–50% of patients after RP during a 10-year follow-up, often without clinical or radiological evidence of disease [3]. It is well known that in 16–35% of cases the patients will receive second line treatments within 5 years from the initial therapy [4]. Freedland et al. found that biochemical recurrence precedes clinical relapse of a median of 5 years and that the time between the end of the therapy and the start of the biochemical failure represents a predictive value for cancer specific survival [5].

At present, according to EAU-guidelines, treatment failure after RP is defined by two consecutive values of PSA higher than 0.2 ng/mL [6]. Once biochemical failure has been diagnosed, it is essential to distinguish between local recurrence and systemic metastases in order to plan the best therapeutic approach. For this reason the clinicians use some parameters that can help to differentiate between local and distant relapse. According to EAU-guidelines there are two specific criteria to theoretically assess the site of tumor recurrence: the rise of PSA over than 0.2 ng/mL within 6–12 months after RP suggests a high risk of local relapse, whereas a PSA increase within a shorter period of time suggests distant metastases. The second criterion used is PSA doubling time (PSAdt) [7]. Several values of PSAdt have been proposed as cut-off to discriminate between local recurrence and systemic metastases. Some authors state that a PSAdt cut-off value lower than 4 months may be more frequently associated with distant metastases, whereas a median PSAdt greater than 12 months predicts local failure [8]. Other papers showed that patients with a PSAdt less than 6 months most probably have systemic metastases, while those with a PSAdt greater than 12 months are more likely to experience local relapse [9].

Other information can be obtained from the pathological examination after RP. The TNM staging system of the International Union Against Cancer recommends to report not only the location but also the extension of extraprostatic invasion because extension is related to the risk of recurrence [10]. For what concerns the surgical margin status, even if there is insufficient evidence to prove a relationship between the extension of positive surgical margins and the risk of relapse [10, 11], surgical margin status is considered an independent risk factor for biochemical failure, particularly for local recurrence.

Treatment of PCa recurrence after RP remains a controversial area and different therapeutic options are available: in the absence of systemic metastases an increase of PSA serum values is supposed to be a locoregional persistent or recurrent disease and salvage radiation treatment could theoretically be the first line treatment. However, if metastatic disease is diagnosed, radiation treatment on the postprostatectomy bed would be unnecessary, with a high risk of morbidity for the patient, and the proper treatment is hormone deprivation therapy [12].

For all the aforementioned reasons there is a strong need for imaging techniques which may be able to recognize small lesions and to identify their nature (persistent or recurrent neoplastic tissue, healthy residual glandular tissue, and granulation tissue or fibrosis). These techniques should be able to detect residual or recurrent disease when the PSA serum level is very low (less than 1 ng/mL) in order to deliver the more relevant therapeutic option as early as possible.

Currently, transrectal ultrasound (TRUS) has neither good sensitivity (Se) nor good specificity (Spe) in detecting early recurrent cancer [13] and TRUS-guided biopsy of the postprostatectomy fossa is not recommended by EAU-guidelines in patients with PSA serum level less than 1 ng/mL. Scattoni et al. showed that TRUS-guided biopsy to detect local relapse after RP has a limited Se (25–54%) when the PSA serum value is less than 1.0 ng/mL [14].

Over the last few years new technological innovations have allowed the development of imaging techniques which link anatomic, functional, and biological information together. Multiparametric Magnetic Resonance Imaging (mp-MRI) and Positron Emission Tomography/Computed Tomography (PET/CT) have proven to be a useful tool in the early diagnosis of PCa recurrence. Both MRI and PET/CT are able to detect subtle changing in cellular metabolism. The spectroscopic imaging shows the relative concentrations of metabolites in the prostatic tissue which are choline, citrate, and creatine. PET/CT is able to highlight cellular metabolism by means of different radiopharmaceuticals: 18F-Fluorodeoxyglucose (18F-FDG), choline (Ch) labeled with 11C or 18F, acetate, and methionine labeled with 11C. The literature shows that Ch is the most useful radiotracer for the detection of PCa cells. The target of metabolic imaging should be to have a good spatial resolution in order to detect very small locoregional recurrences in patients with biochemical relapse and very low values of PSA.

To the best of our knowledge, both PET/CT and mp-MRI have provided promising results in the detection and localization of local PCa recurrence after RP.

## 2. Hybrid Imaging: PET/CT

PET is a molecular imaging that is able to create images from physiological and metabolic processes. PET uses positron emitters to create quantitative tomographic images. PET images are volumetric set of data that can be displayed as tomographic images in the transaxial, coronal, or sagittal planes. At the same time PET has a limitation: the lack of an anatomical reference frame. For this reason the combination of PET with CT offers optimal fusion of images with an excellent morphological imaging with anatomical resolution.

PET/CT is a molecular imaging hybrid technique that combines, in a single whole body session, metabolic and functional information of oncologic diseases with anatomic information provided by CT component of the exam. Fused PET/CT images can be assessed in multiparametric modality in axial, sagittal, or coronal planes, thanks to dedicated soft wares.

Malignant cells are usually characterized by a higher glucose metabolism compared with benign ones. Increased 18F-FDG uptake is correlated with increased cellular proliferation. For this reason the most used radiotracer is the 18F-FDG, an analogue of glucose.

In the case of PCa, 18F-FDG tracer, however, is of a limited use in the diagnosis, staging, and follow-up because of urinary excretion and low uptake by prostatic parenchyma. The physiological concentration in the bladder implies a difficult evaluation of the prostate bed. It has also been documented that the uptake of the 18F-FDG in the primary tumor of the prostate gland and in bone metastases may be reduced: the diagnostic Se of 18F-FDG PET in detecting skeletal metastases ranges between 18 and 65%, significantly lower than the sensitivity of bone scan. This fact is probably attributable to the slow proliferation of PCa [15, 16]. Sanz et al. reported similar findings in 1999, with 18F-FDG PET unable to reliably detect lymph node metastases [17]. 18F-FDG has been estimated to detect nodal metastases with a Se ranging from 0 to 50% and a Spe ranging from 72 to 90% [18].

Carcinogenesis is characterized by an increase of cell proliferation and, therefore, by means of induction of Ch kinase activity and by an increase of phospholipids in the neoplastic tissue. Hara et al. [19] on the basis of these observations introduced a new radioactive tracer in diagnosing of cancer: the methyl-Ch labeled with carbon-11 (11C-Ch), a substrate for the synthesis of phosphatidylcholine.

This radiotracer has been employed in various tumours, such as cerebral [20], lung [21], and bladder [22] tumors. As demonstrated by numerous studies published in recent years, its main clinical use is, however, the study of PCa, since PCa is a malignant neoplasia but characterized by very low glucose metabolism. The 11C-Ch, in fact, is taken up in the pelvis exclusively by prostatic tissue and this property is retained by the neoplastic tissue. In addition, this radiopharmaceutical has a negligible clearance through the urinary system.

The accuracy of Ch-PET/CT in staging and restaging of PCa has been evaluated by several studies [23, 24].

As regards 18F-FDG, some studies showed that Ch-PET/CT can detect a larger number of metastatic lymph nodes and bony metastatic lesions than 18F-FDG PET/CT in patients with PCa [25]. Furthermore, Picchio et al. found that Ch-PET/CT, compared to 18F-FDG PET/CT, can identify a greater number of local recurrences (42% versus 27%) and showed that Ch-PET is more accurate in identifying both local and distant relapses [26].

**Figure 1:**
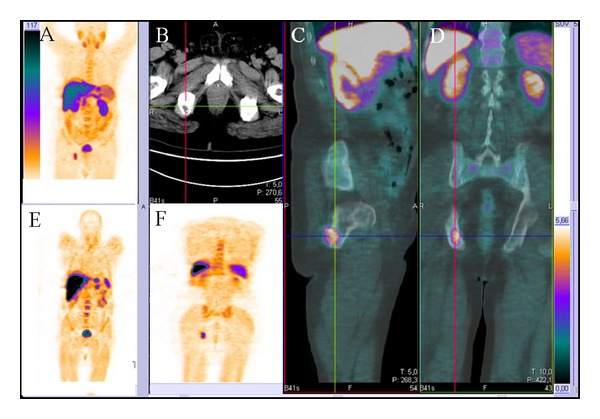
Ch-PET/CT images of a 73-year-old patient with biochemical failure (PSA serum value 2.1 ng/mL) after radical prostatectomy for prostate cancer with a metastatic lesion at the right ischiopubic branch. (A, E, and F) Whole body coronal PET images showing an uptake of the radiotracer at the level of the right ischiopubic branch. No uptake at the lymph node stations and at the level of the postprostatectomy bed was found. (B) Axial morphological CT image of the pelvis displaying a hypodense nodular lesion at the level of the right ischiopubic branch. (C) Sagittal and (E) coronal fused PET-CT images showing the uptake of the radiotracer at the level of the right ischiopubic branch.

There are several papers about the role of Ch-PET in primary PCa detection [27, 28] and its role in staging prostatic disease before treatment [29–32]. However, since the Ch uptake can occur in some benignant conditions, such as prostatitis or prostatic hyperplasia, the role of this technique in this field is still not well clear. Restaging of PCa is, therefore, the main field of Ch-PET/CT imaging. In particular, its main role is in identifying the site of recurrent disease in patients who present with biochemical failure after RP (Figure 1).

The 11C-Ch is characterized by a short half-life (approximately 20.4 minutes), and for this reason its use is allowed only in centers provided with a cyclotron. In consideration of the logistical limitations of the use of 11C-Ch, Ch was subsequently labeled with 18F, which, thanks to the increased half-life (109.8 min), allows storage and transport. However, 18F-Ch radiotracer is characterized by an increased urinary excretion compared to 11C-Ch.

Heinisch et al., in a single-centre retrospective study, analyzed 31 men after RP and found that 8/17 patients (47%) with biochemical failure and with a PSA serum value less than 5 ng/mL had positive results at 18F-Ch PET-CT examination. Furthermore they found malignancy in 7/8 patients confirmed by either biopsy or by the subsequent course of the disease [33].

Rinnab et al., in a single-centre retrospective study, analyzed 50 men with biochemical relapse after RP for PCa (average PSA serum value: 3.62 ng/mL; range 0.5–13.1 ng/mL); the authors considered the Se and Spe of 11C-Ch PET-CT only in patients with PSA serum level lower than 2.5 ng/mL, reporting 91% and 50%, respectively [34]. In another single-centre retrospective study Rinnab et al. enrolled 41 patients with biochemical recurrence following RP (average PSA value was 2.8 ng/mL; range 0.41–11.6 ng/mL): overall the Se of 11C-Ch PET-CT was 93%, Spe 36%, positive predictive value (PPV) 80%, and negative predictive value (NPV) 67% [35].

Castellucci et al. enrolled 190 men with biochemical failure after RP (mean PSA serum level 4.2 ng/mL; range 0.2–25.4 ng/mL) and found an overall Se of 11C-Ch PET-CT of 73% and Spe of 69% [36]. The same authors, in another single-centre retrospective study, analysed 102 patients with biochemical recurrence after RP (PSA serum level ranging from 0.2–1.5 ng/mL) who underwent 11C-Ch PET-CT scan; all suspected local recurrences at PET-CT exam were confirmed afterwards by TRUS-guided biopsy [37]. Regarding local recurrence the Se of 11C-Ch PET-CT was 53.8% and Spe was 100% (no false positive was recorded). Giovacchini et al. in a single-centre retrospective study analysed 170 patients (presenting with biochemical relapse after RP) with Ch-PET-CT; the average PSA serum value at the time of the exam was 3.24 ng/mL (range 0.23–48.6 ng/mL) and mean PSAdt was 9.37 months [38]. Ch-PET-CT showed a Se of 87%, Spe of 89%, PPV of 87%, NPV of 89%, and accuracy of 88%. In this work, Ch-PET-CT positive findings were confirmed using one of the following methods: histological analysis of lymph node specimen, biopsy of the urethra/bladder neck anastomosis, progression on PET-CT follow-up studies associated with increased PSA serum level, confirmation with conventional imaging, disappearance or sizable reduction of Ch uptake after local or systemic treatment, and PSA decrease greater than 50% after selective irradiation of the unique site of pathological Ch uptake.

In another second single-centre retrospective study, Giovacchini et al. enrolled 358 men with biochemical failure after RP: the average PSA serum value was 3.77 ng/mL (range 0.23–45.2 ng/mL). 11C-Ch PET-CT was performed in all patients and results were validated by histological analysis. The authors concluded that 11C-Ch PET-CT had an overall Se of 85%, Spe 93%, PPV 91%, NPV 87%, and accuracy 89% [39].

In a third single-center retrospective study Giovacchini et al. from a database of 2124 patients retrospectively analysed 109 men with biochemical relapse (average PSA before imaging of 1.31 ng/mL, range 0.22–16.76 ng/mL) who underwent 11C-Ch PET-CT. They reported positive findings at 11C-Ch PET-CT in 12/109 patients, which were confirmed subsequently to be local recurrence in 4 patients and pelvic nodal metastases in 8 cases [40].

A considerable limit of all these studies is the lack of information on the dimensions of local recurrence.

Reske et al. in a single-centre retrospective study, analysed 49 men with average PSA serum level of 2 ng/mL and median maximal diameter of the lesions of 1.7 cm (range 0.9–3.7 cm), who underwent 11C-Ch PET-CT scan. TRUS-guided biopsy was used to validate the results. They found a Se of 73%, Spe 88%, PPV 92%, NPV 61%, and an accuracy of 78% [41].

For the study of PCa the acetate labeled with 11C has also been proposed, which appears promising as it accumulates in the cells proportionally to the biosynthesis of fatty acids (in particular phospholipids), which is increased in neoplastic tissue. 11C-acetate has been proposed by some authors to be more sensitive for the detection of local nodal metastases and has had mixed results when compared with 18F-FDG for the detection of bone metastases [42–44]. Albrecht et al. demonstrated that this radiotracer could detect local recurrence in five of six patients [45]. Vees et al. [46] in a multicentre retrospective study, evaluated 20 patients divided into two different groups with biochemical recurrence or suspected residual tumour after RP with a PSA serum level less than 1.0 ng/mL (range 0.11–0.73 ng/mL): in the first group they used 18F-Ch PET-CT to detect local recurrence whereas in the second group they used 11C-acetate PET-CT. They reported a Se of 60% and 66% for 18F-Ch PET-CT and 11C-acetate PET-CT, respectively, in detecting local relapse. Sandblom and coworkers have also demonstrated the ability of 11C-acetate to detect local recurrence rate with 75% Se and a false-positive rate of 15% [47]. Other PET tracers under study are the 18F-dihydrotestosterone (18F-DHT) that binds to androgen receptors, the 11C-methionine, a marker of protein synthesis, and the 18F-fluoride that accumulates in areas of increased bone turnover are used for the depiction of skeletal metastases and demonstrate improved sensitivity and specificity compared with planar and single-photon emission computed tomography (SPECT) for the detection of osseous metastases [48]. It remains to be seen if this tracer will gain cost effectiveness and widespread use.

Up to now the overall Ch-PET/CT Se in detecting sites of PCa locoregional recurrence ranges between 38% and 98%. Moreover it has been demonstrated that Ch-PET/TC positive detection rate improves with increasing PSA serum values.

The most significant information provided by all the cited studies on this topic is about the apparently very tight relationship between Ch-PET/CT detection rate and PSA serum level in restaging patients with PCa. In the last decade, several authors proposed some cut-off values of PSA serum level to help in identifying those patients who can potentially derive a benefit by a Ch-PET/CT examination. Cimitan et al. proposed that a PSA cut-off value higher than 4 ng/mL is more likely to be associated with a greater chance to detect systemic metastases [49].

It has been found that the higher the value of PSA at the time of the Ch-PET/CT examination is, the greater the detection rate of Ch-PET/CT: 36% for values of PSA less than 1 ng/mL; 43% for PSA values between 1 and 2 ng/mL; and 62% for PSA values between 2 and 3 ng/mL and 73% if the PSA is higher than 3 ng/mL [50].

More recently, several authors proposed lower PSA cut-off values to individuate patients that could benefit from a Ch-PET/CT scan. Rinnab et al. proposed a cut-off value of 1.5 ng/mL but, generally, various authors agree that the exam has better Se when performed in patients with PSA serum level higher or equal to 2 ng/mL [34, 35, 38].

Krause et al. have demonstrated that the Ch-PET-CT examination is a very useful tool, even when the value of PSA is less 1 ng/mL, in differentiating only locally confined disease by metastatic disease, with significant implications for the clinical management of the patient: (1) local therapy is not appropriate if distant locations are proved, (2) the local treatment of second line has greater success in the case of low PSA values, and (3) the choice of therapeutic strategy can be customized in case of abdominal and pelvic lymph nodes involvement (radiation therapy and/or systemic therapy) [50].

More recently, many investigators have focused their attention on the potential role of PSA kinetics such as PSAdt and PSA velocity (PSAvel), which is a PSA derivative determined as linear regression of the PSA values over time [9].

In particular, as reported by the literature data, PSAdt and PSAvel values are correlated with specific mortality risk of PCa [51]. In addition, it has been well demonstrated that the risk of systemic metastases in patients with biochemical relapse after RP depends on PSA serum levels and PSAdt values. Notably, it has been shown that, when PSAdt is longer than 6 months, the risk of metastasis is less than 3%, even if absolute PSA values are more than 30 ng/mL. If PSAdt is shorter than 6 months and PSA is more than 10 ng/mL, the risk of metastasis is about 50% [52].

Partin et al. evaluated the capability of PSAvel in predicting relapse after RP and found that it turns out to be of some help to combine data relative to PSAvel, Gleason score, and pathological staging in differentiating locoregional recurrence from distant metastases [53].

Generally, the Se of Ch-PET/CT is significantly higher in patients with a PSAvel higher than 2 ng/mL/year or a PSAdt lower than 6 months [54]. The proposed PSAvel cut-off of 2 ng/mL/year seems to be the more reliable value to distinguish patients with a positive Ch-PET/CT examination from those with a negative scan in a more accurate way, even if there are some authors who suggest that patients with a PSAvel higher than 1 ng/mL/year could benefit by a Ch-PET/CT scan [55].

Rybalov et al. found a detection rate of 11C-Ch PET/CT lower than 50% in patients with total PSA lower than 2 ng/mL and/or PSAvel lower than 1 ng/mL/year. Marzola et al. in a patient population of 233 men with biochemical recurrence after RP found a detection rate of 18F-Ch PET/CT of 54%, which significantly increases with the increase in trigger PSA [56, 57].

In a recent meta-analysis Treglia et al. reported that the pooled detection rate of radiolabelled Ch-PET/CT in restaging PCa was 58% [95% confidence interval (CI) 55–60] and they found that it increased to 65% (95% CI 58–71) when PSAdt was lower than 6 months and to 71% (95% CI 66–76) and 77% (95% CI 71–82) when PSAvel was greater than 1 or 2 ng/mL/year, respectively [58]. PSAdt lower than 6 months and PSAvel more than 1 or 2 ng/mL/year proved to be relevant factors in predicting the positive result of radiolabelled Ch-PET/CT.

In all cited studies the very good detection rate and the Se of Ch-PET/CT scan are often associated with distant metastases (both bone metastases or lymph nodes) while the available data about the depiction of locoregional recurrence are still discordant. In particular, in the studies where the mean PSA serum level is lower than 1.5 ng/mL, the detection rate of Ch-PET/CT for local relapse is doubtless poor, probably because of low PET spatial resolution (5-6 mm) which limits the detection of small lesions. In a recent study Hausmann et al. assessed the possibility of using time-of-flight (TOF) reconstruction algorithm to obtain an increase of spatial resolution to detect small metastatic lesions and local recurrence. They concluded that TOF seems to be of additional value to detect small metastatic lesions in patients with PCa and biochemical recurrence, which may have further clinical implications for secondary treatment [59].

Another limitation of 11C-Ch PET/CT is represented from any finding of false-positive results due to the presence of accumulation of tracer in some benign prostate disease and in case of reactive lymph nodes (where it accumulates in granulocytes and macrophages).

Most studies state that the routine use of Ch-PET/CT for localization of locoregional recurrence of PCa cannot be recommended for PSA serum values less than 1 ng/mL [60, 61]. However in a more recent study Mamede et al. investigated the role of Ch-PET/CT in patients with biochemical relapse after RP showing PSA values lower than 0.5 ng/mL and found that Ch-PET/CT can be used even if PSA values are very low, preferentially in hormonal resistant patients showing fast PSA kinetics [62].

An early detection of the site of relapse could lead to a personalized and tailored treatment; for example, PET/CT fused images might be useful for delineating local sites of recurrence within the prostatic resection bed allowing a boost to PET positive sites (in addition to conventional local irradiation of the prostatic fossa) [63].

In summary, in agreement with the literature data, Ch-PET/CT could play a role in managing patients with PCa, in particular during the restaging phase, thanks to its good Se with regard to systemic metastases and good detection rate in relationship with PSA serum value higher than 1 ng/mL, PSAdt lower than 6 months, and PSAvel higher than 2 ng/mL/year. To date, its role in detecting locoregional recurrence in postprostatectomy fossa after radical surgical treatment still remains unclear in patients with biochemical failure and low PSA values.

## 3. Multiparametric MRI

Over the last 20 years substantial progress has been made in MRI clinical employment. High-field strength endorectal coil MRI is able to produce morphological T2-weighted imaging of the prostate gland. Among the other recent complementary functional techniques that improve both staging and detection of PCa there are dynamic contrast-enhanced MRI (DCE-MRI), 1H-spectroscopic imaging (1H-MRSI), and diffusion-weighted imaging (DWI) [64, 65]. DCE-MRI is a technique that can detect those tumors in which an angiogenic pathway has been turned on, as it is based on Gradient-echo T1-weighted sequences which can assess neoangiogenesis during the passage of a gadolinium contrast agent [66].

DWI provides qualitative and quantitative information about cellularity density of the tissue and cell membrane integrity. Intracellular and extracellular water molecules move freely in all directions. In neoplastic prostatic tissue extracellular space is decreased; therefore the movement of water molecules is restricted and the so-called apparent diffusion coefficient values are low compared to healthy prostatic tissue. DWI can be performed without the administration of exogenous contrast medium and it does not require long acquisition times and therefore it can be considered the functional technique more practical and simple to use. MRSI provides three-dimensional data set of the prostate gland, with volume voxels ranging from 0.24 cm to 0.34 cm [67]. This functional technique evaluates the relative concentration of metabolites within voxels, such as citrate, choline, and creatine. Previous studies demonstrated that citrate levels are reduced in PCa tissue; on the contrary creatine and particularly choline are increased. The peak integral ratio of choline plus creatine to citrate can distinguish PCa tissue from healthy glandular tissue [67]. Conforming to the literature each voxel can be defined as follows: fibrotic or scar tissue when the ratio is less than 0.2, residual healthy prostatic glandular tissue when the ratio is between 0.2 and 0.5, probably recurrent PCa when the ratio is between 0.5 and 1, and definitely recurrent PCa tissue when the ratio is higher than 1 [68]. Compared with DWI or DCE-MRI, MRSI is a more complex functional technique and it also requires longer acquisition times.

The main advantage of Mp-MRI is its very good spatial resolution which allows the localization and characterization of PCa tissue and the detection of very small lesions and it is also able to better differentiate healthy glandular tissue from neoplastic zones. It is a complex exam and it needs therefore experienced and trained radiologists, in particular if MRSI is performed.

**Figure 2:**
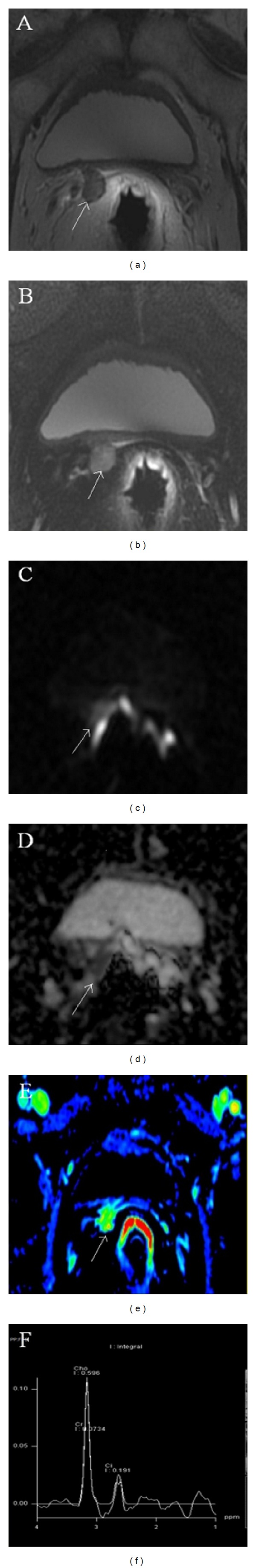
Multiparametric-MR images of a 71-year-old man with prostate-specific antigen progression (PSA serum level 0.47 ng/mL) after radical retropubic prostatectomy, with suspected local recurrence. (a) Axial T2-weighted fast spin-echo and (b) axial T2-weighted fat saturated fast spin-echo images show, on the zone previously occupied by the right seminal vesicle, a slightly hyperintense lobulated tissue (white arrow). (c) Axial native DWI image with *b* value of 1000 s/mm^2^and (d) ADC map reconstructed from images obtained at *b* values of 0, 500, and 1000 s/mm^2^ show a dark area corresponding to the abnormal hyperintense tissue seen on T2-weighted images. (e) Gradient-echo T1-weighted color map image shows a well-defined area of marked enhancement (white arrow) on the same location as the nodular tissue seen on T2-weighted images. (f) 1H-magnetic resonance spectroscopic imaging reveals a high choline peak with a choline-plus-creatine-to-citrate ratio greater than 1. All these findings are consistent with local recurrence.

Recently, mp-MRI more than other imaging techniques (Figure 2) has been proposed as a useful tool in the diagnostic process of local recurrence of PCa after RP [69].

Once biochemical progression of PSA serum values occurs after RP; it is essential—for treatment planning—to determine whether the relapse is at local or distant sites; moreover the possibility of residual glandular healthy tissue should be taken into account as well. Given this scenario, diagnostic imaging techniques are very useful to distinguish locoregional cancer recurrence from systemic relapse and therefore to refer patients to the best therapeutic approach (i.e., radiation therapy for local recurrence and hormone deprivation therapy for systemic disease) [12]. Moreover, it is very important for radiation oncologists to differentiate a residual glandular healthy tissue from a locoregional neoplastic recurrence because the dose of radiation therapy delivered in the postprostatectomy fossa is quite different [70].

Currently Ch-PET/CT is recommended when PSA serum level is higher than 1 ng/mL because this technique has good Se and Spe in detecting metastatic lymph nodes, distant metastases, and local neoplastic recurrences after RP only in patients with high PSA serum values. In addition, the accuracy of Ch-PET/CT to detect locoregional PCa recurrences relies on the size of the lesion, being usually higher if the lesion is more than 1 cm in diameter [40, 41].

Although Ch-PET/CT is recommended in patients with high PSA serum values, in patients who experience low biochemical alterations after RP (PSA serum value between 0.2 and 1 ng/mL) it is very important to exclude the presence of locoregional recurrence, with this information being essential for radiation oncologists.

Up to now within mp-MRI DCE-MRI is assumed as the most reliable technique in detecting locoregional neoplastic recurrence [68, 71].

Presently there are several studies which demonstrate the usefulness of mp-MRI in depicting locoregional cancer relapse. Mp-MRI after RP is currently indicated to detect small locoregional relapse and also to discriminate between residual glandular healthy tissue, fibrotic tissue, granulation tissue, and nodule recurrence. It may also be able to assess the aggressiveness of nodule recurrence. Panebianco et al. compared ADC values of locoregional recurrences with the histological results [72]. The average and standard deviation of ADC values were 0.5 ± 0.23 mm^2^/s for high-grade aggressiveness relapse, 0.8 ± 0.09 mm^2^/s for intermediate-grade aggressiveness relapse, and 1.1 ± 1.17 mm^2^/s for low-grade aggressiveness recurrence; ADC values higher than 1.3 mm^2^/s (mean ADC values 1.4; range 1.3–1.7) were found in patients with a histological finding of residual glandular healthy tissue.

Perianastomotic scar/fibrotic changings appear hypointense on T2-weighted images without enhancement on DCE-MRI. On the other hand both benign and recurrence nodules appear as tissues with intermediate to high signal intensity on T2-weighted morphological images compared to pelvic muscles and show enhancement after intravenous injection of contrast medium. On DCE-MRI images all benign nodules show signal enhancement less than 50% in the early phases, whereas neoplastic recurrence tissues display fast and avid signal enhancement in the early phases followed by a plateau or washout on the signal/intensity curve.

Sella et al. in a single-centre retrospective study analyzed 48 men with biochemical failure after RP with an average lesion size of 1.4 cm (maximum diameter ranging from 0.8 to 4.5 cm) and a mean PSA serum value of 2.18 ng/mL (range 0–10 ng/mL); the Se and Spe of mp-MRI were, respectively, 95% and 100%, but they were reached with a small group of patients and with a very large size of local relapse and a very high PSA serum value [73].

Further studies confirmed the importance of mp-MRI in detecting local PCa recurrence in patients with biochemical relapse after RP.

Cirillo et al. in a patient population of 72 men (local recurrence maximum diameter ranging from 0.8 to 3.5 cm, mean maximum diameter 1.7 cm, average PSA serum value 1.23 ± 1.3 ng/mL, and range 0.2–8.8 ng/mL) compared T2-weighted to DCE-MRI images and they found a sensitivity, specificity, and accuracy of 61.4%, 82.1%, and 69.4% for T2-weighted images and 84.1%, 89.3%, and 86.1% for DCE-MRI images [74].

Casciani et al. in a single-centre retrospective study evaluated the role of mp-MRI with DCE in 46 men who previously underwent RP (average maximum diameter of the local recurrence 1.5 cm, ranging from 0.4 to 4.0 cm, and mean PSA serum value 1.9 ng/mL, ranging from 0.1 to 6.0 ng/mL) and they obtained a Se, Spe, and accuracy of 88%, 100%, and 94%, respectively [71].

Although these studies were based on a sizeable number of patients and the average PSA serum value was not very high, their accuracy is partially limited by the mean size of locoregional relapse which is always higher than 1.5 cm.

Sciarra et al., in a population of 70 patients (average PSA serum value in group A 1.26 ng/mL and in group B 0.8 ng/mL; mean size of the lesions 13.3 mm in group A and 6 mm in group B), compared 1H-MRSI and DCE-MRI and they found a sensitivity of 71–84% and a specificity of 83%–88% for 1H-MRSI alone, a sensitivity of 71–79% and a specificity of 94–100% for DCE-MRI alone, and for the two combined techniques a sensitivity of 86–87% and specificity of 94–100% [68].

In a recent study Panebianco et al., in a population of 84 men (average PSA serum level 1.1 ng/mL in group A and 1.9 ng/mL in group B; mean size of the lesions 6 mm in group B and 13.3 mm in group B), found that a combined technique of 1H-MRSI and DCE-MRI at 3 Tesla magnet is a valid tool to detect locoregional PCa relapse and it is more accurate than Ch-PET/CT in the identification of small lesions in patients with low biochemical progression after RP (PSA serum values ranging from 0.2 to 2 ng/mL) [75].

These last two recent studies were based on a considerable number of patients and detected tumor relapses less than 1.5 cm in maximum diameter, but they did not compare DCE-MRI with DWI technique.

Nowadays there is an increasingly growing interest in the use of DWI because it is an emergent, noninvasive technique that can be acquired without the intravenous injection of contrast medium, and it does not require long acquisition times and therefore it can be considered the functional technique more practical and simple to use [76]. It is a powerful tool in detecting and localizing PCa recurrence in patients with biochemical progression after definitive radiation therapy [77, 78]. In a recent study Giannarini et al. described five patients with biochemical failure after RP and pelvic lymph node dissection in whom locoregional relapse could only be depicted with DWI [79]. Recent studies demonstrate the usefulness of DWI technique as a valid toot in detecting local cancer recurrence after external beam radiation therapy [77] and after RP [78], even though these early experiences were based on a small number of patients.

**Figure 3:**
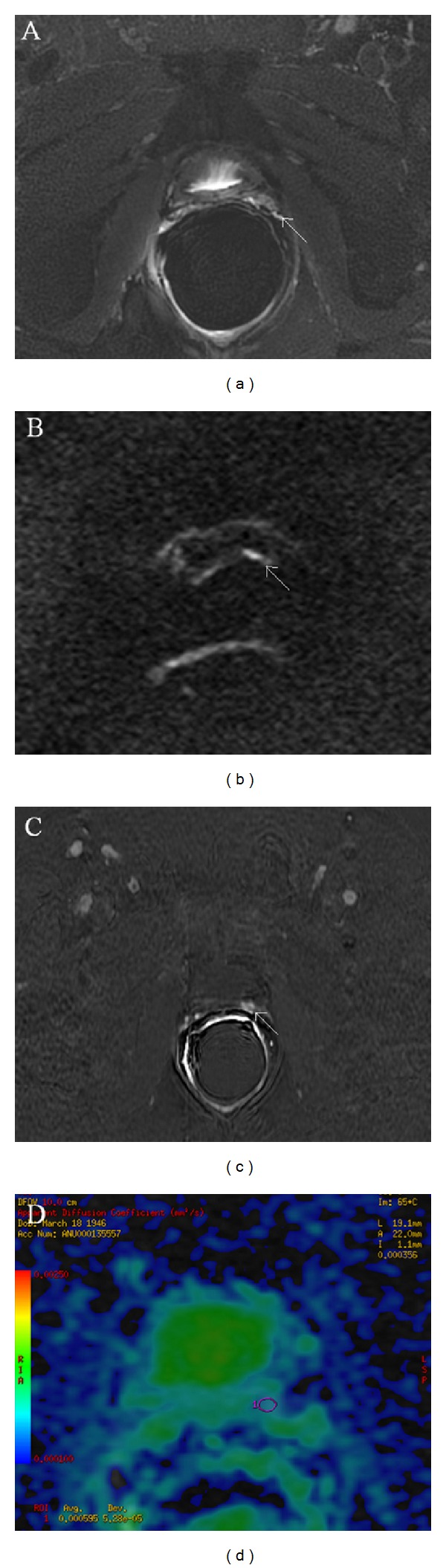
Multiparametric-MR images of a 71-year-old man with prostate-specific antigen progression (PSA serum value 0.6 ng/mL) after radical retropubic prostatectomy, with suspected local recurrence. (a) Axial T2-weighted fat saturated fast spin-echo image shows, on the zone previously occupied by the left seminal vesicle, a hyperintense solid nodular tissue (white arrow) compared to pelvic muscles, of about 7 mm in size. (b) Axial DWI image with a *b* value of 1000 s/mm^2^ shows a focal area of restricted diffusion (white arrow) corresponding to the solid nodular tissue detected on T2-weighted image. (c) Axial Gradient-echo T1-weighted subtracted image showing a remarkable enhancement of the pathological tissue. All these findings are consistent with locoregional relapse. (d) ADC map reconstructed from images obtained at *b* values of 0, 500, and 1000, where the ROI was plotted for the measurement of ADC values in order to assess the aggressiveness of the nodule.

Panebianco et al. in a single-centre prospective study analyzed a large number of men (262 consecutive male patients) in order to validate the role of 3 Tesla DWI in mp-MRI in the detection of local PCa relapse in patients with biochemical failure after RP [72]. All in all the accuracy of DWI was slightly lower than DCE (92% versus 93% in group A and 89% versus 91% in group B). The authors suggested that the overall accuracy of DCE imaging is superior to that of DWI because DW images are more affected by intrinsic distortion artifacts and background noise than DCE images are, though there are some cases in which DCE is quite doubtful and DWI is needful for PCa local recurrence detection. As an example, sometimes it is very difficult to distinguish between a prominent periprostatic venous plexus and an enhancing recurrent nodule on the basis of DCE alone [80]; therefore when there is this potential pitfall DWI is a very useful technique to exclude the presence of pathological tissue in postprostatectomy fossa (Figure 3). This experience points out to the diagnostic power of DWI which is a functional technique nearly comparable to DCE-MRI, therefore justifying a MRI protocol of postprostatectomy bed composed only of morphological T2-weighted images and DWI images in patients with renal failure. Moreover, these results could pave the way to the possibility of using DWI as an alternative functional technique to DCE-MRI for follow-up of patients with biochemical progression after RP, with a Se, Spe, and accuracy in depicting local relapse almost comparable to those of DCE-MRI. In addition, the possibility of using DWI as an alternative functional technique to DCE is considered thanks to its short acquisition time and repeatability, which are superior to those of DCE, and also because DWI, given the absence of intravenous administration of contrast medium, is free from complications and danger.

The depicting of local recurrence nodule by means of Mp-MRI is, moreover, of primary importance if external beam salvage radiation therapy is scheduled and feasible. Salvage external beam radiation therapy consists, typically, in the irradiation of the whole postprostatectomy bed with a delivered dose of 64/68 Gy in 2 Gy per daily fraction 5/week [81].

In this setting whenever Mp-MRI examination displays the locoregional relapse, it is possible to deliver to the pathological tissue discovered a radiation boost above 70 Gy with a shrinked field or with simultaneous integrated boost or even with SRT (stereotaxis radiation therapy) approach [82].

This specific treatment, that is a dose adapted MRI based approach treating the prostate bed with a boost to local recurrence, may also potentially improve the therapeutic ratio by selecting patients that are most likely expected to benefit from higher radiation doses. This kind of approach can furthermore improve the control of local disease avoiding further locoregional relapses over time. Moreover, the most recent radiation therapy techniques such as Intensity-Modulated Radiation Therapy with IGRT (image-guided radiation therapy) and Volumetric Modulated Arc Therapy allow both a lower toxicity to normal surrounding tissues (bladder, rectum, anal canal and penile bulb, and head of the femoris) and a more focused irradiation of the recurrence nodule without possibility of geographic miss and give us also the chance of utilization of ipofractionated radiation therapy regimen and dose escalation [83].

The new hybrid PET/MRI scanners, with simultaneous acquisition of mp-MRI and PET images, can provide combined structural, metabolic, and functional imaging information that can potentially be of some important benefit for patient management and outcome [84]. In a recent review by Thorwarth and Leibfarth on the potential role of PET/MRI in radiation therapy, the authors found that Ch-PET/MRI might be of value for target volume delineation of primary and recurrent prostate cancer as well as in the identification of prostate cancer lymph node involvement. The authors concluded that hybrid PET/MRI might improve radiation treatment planning by enabling more precise target volume delineation and also might provide a basis for dose painting [85].

## 4. Final Considerations

Currently, Ch-PET/CT is the most promising whole body imaging modality in detecting distant metastases of PCa, because of its ability to depict small pathological lymph nodes and bone metastases with a high sensitivity, specificity, and accuracy. This feature is of primary importance on management of patients with PCa and for evaluating their prognosis, thanks to the possibility to assess in a single session both anatomic and metabolic information about the disease.

This role can be heightened by the relevant selection of the patients treated with surgical curative intent by means of biochemical markers such as PSA serum values and PSA kinetics (PSAdt and PSAvel). Furthermore, the concurrent hormonal deprivation therapy has to be taken into account because it could negatively affect the Se of the exam. To date, the role of this diagnostic tool in detecting local recurrence in postprostatectomy bed after RP in patients with biochemical failure and low PSa values still remains unclear. The detection rate of Ch-PET-CT for locoregional relapse seems to be poor, probably because of limited spatial resolution (5-6 mm) of PET scanners which does not allow the depiction of small lesions.

Recently, mp-MRI has been proposed, more than other imaging procedures, as a useful tool in the diagnostic process of local recurrence of PCa after RP. Currently mp-MRI after RP is indicated to diagnose small local cancer recurrence in a range of PSA serum values between 0.2 and 1 ng/mL when Ch-PET/CT is not eligible. Moreover Mp-MRI, thanks to functional techniques, allows distinguishing between residual glandular healthy tissue, scar/fibrotic tissue, granulation tissue, and tumour recurrence and it may also be able to assess the aggressiveness of nodule recurrence. In addition an MRI based radiation therapy approach treating the prostatic fossa with a boost to local recurrence improves the treatment therapeutic ratio and allows a decrease of locoregional relapses.

Further studies are needed to evaluate the ability of Ch-PET/CT in the detection of locoregional PCa recurrence. Moreover, the recent development of hybrid PET/MRI scanners could improve the diagnostic accuracy in depicting local PCa relapses in postprostatectomy fossa.
